# Subsequent vertebral fracture after percutaneous vertebroplasty or kyphoplasty in postmenopausal women with osteoporotic vertebral compression fracture

**DOI:** 10.3389/fendo.2026.1716002

**Published:** 2026-02-12

**Authors:** Qianru Zhang, Yuanpei Cheng, Fengling Chen

**Affiliations:** 1 Department of Endocrinology and Metabolism, Shanghai Ninth People’s Hospital, Shanghai Jiao Tong University School of Medicine, Shanghai, China; 2 Department of Orthopedics, China-Japan Union Hospital of Jilin University, Changchun, China

**Keywords:** osteoporotic vertebral compression fracture, percutaneous kyphoplasty, percutaneous vertebroplasty, postmenopausal women, subsequent vertebral fracture

## Abstract

**Background:**

Subsequent vertebral fracture (SVF) as a severe complication of percutaneous vertebroplasty (PVP) or percutaneous kyphoplasty (PKP) for the treatment of osteoporotic vertebral compression fracture (OVCF) is a major public health challenge. Risk factors of SVF following initial PVP or PKP surgery in postmenopausal women are still controversial. This study aims to investigate the risk factors of SVF after initial PVP or PKP for single-level OVCF in postmenopausal women.

**Methods:**

Postmenopausal women who were initially treated with PVP or PKP for single-level OVCF were retrospectively analyzed. The patients were divided into SVF group and control group based on whether they had SVF or not. Demographic, surgery-related and radiographic data were recorded from electronic medical records or radiographic examinations. Univariate and multivariate logistic regression analysis were conducted to determine the independent risk factors.

**Results:**

A total of 528 postmenopausal women who met the inclusion and exclusion criteria were included in this study, and 73 postmenopausal women experienced SVF after PVP or PKP. Logistic regression analysis demonstrated that low BMI (OR = 0.905; 95% CI = 0.829-0.987, *P* = 0.025), previous fracture history (OR = 1.899; 95% CI = 1.046-3.449, *P* = 0.035) and low BMD (OR = 0.977; 95% CI = 0.966-0.988, *P* < 0.001) were correlated with SVF following initial PVP or PKP for single-level OVCF in postmenopausal women.

**Conclusions:**

Low BMI, previous vertebral fracture history and low BMD were independent risk factors of SVF in postmenopausal women treated with initial PVP or PKP for single-level OVCF.

## Introduction

Osteoporosis is a common metabolic disorder with the characteristic of low bone mineral density (BMD), increased bone fragility and high susceptibility to fracture (1). Most commonly, it occurs in postmenopausal women due to estrogen deficiency (2, 3) and is considered to be a common musculoskeletal disease of aging. Osteoporotic vertebral compression fracture (OVCF) is the most common fracture secondary to osteoporosis in the populations aged 50 years old and above, with the prevalence of 20%-24% (4). OVCF can lead to severe pain, disability, kyphosis, decreased quality of life, and even mortality (5–7).

It is well established that conservative treatments including analgesia agent, bed rest and bracing have proven to be effective in treating OVCF. Minimally invasive surgery, including percutaneous vertebroplasty (PVP) and percutaneous kyphoplasty (PKP), is recommended for the treatment of OVCF in patients who do not response to conservative treatments, which has good safety and effectiveness reported by several studies (8–10). However, some randomized controlled studies reported that no significant differences observed between the PVP or PKP group and controls (11, 12). A controversy exists regarding the efficacy of PVP or PKP in treating OVCF. Most seriously, subsequent vertebral fracture (SVF) after PVP or PKP has gained increasing concern and become an issue that cannot be ignored (13–15).

Recently, accumulating evidence has indicated some risk factors of SVF after PVP or PKP in treating OVCF (16–19). However, no study has been reported on the risk factors of recurrent fractures following initial PVP or PKP for the treatment of single-level OVCF in postmenopausal women. More seriously, little attention has been paid to the prevention of SVF. It is necessary to identify and detect the potential risk factors of SVF after initial PVP or PKP, especially for postmenopausal women with high risk. We hypothesized that body mass index (BMI), BMD, and previous vertebral fracture history are independent risk factors of SVF after initial PVP or PKP in postmenopausal women. Therefore, the research aimed to investigate the risk factors of SVF and provide orthopedic surgeons with evidence-based guidance in clinical practice, which may help to reduce or even avoid the incidence of SVF in postmenopausal women who underwent PVP or PKP for single-level OVCF.

## Methods

### Participants

Postmenopausal women who were treated with initial PVP or PKP for the treatment of single-level OVCF between October 1, 2019 and February 28, 2022 in the hospital were enrolled in this study. It is should be noted that SVF is defined as a new vertebral compression fracture following the initial treatment of an OVCF by PVP or PKP, based on the presence of new back pain and corroborating radiographic evidence. Patients with SVF were categorized into SVF group, whereas those without SVF were divided into control group. The inclusion criteria were as following: (1) postmenopausal women aged 50 years and over; (2) back pain due to OVCF; (3) single-level OVCF; (4) initial PVP or PKP; (5) low or minimal trauma; (6) complete data; (7) the follow-up of at least 1 year. The exclusion criteria were as following: (1) initial multi-level OVCFs treated with PVP or PKP; (2) vertebral burst fracture due to high-energy trauma; (3) incomplete posterior wall of vertebral body; (4) neurological symptoms; (5) pathological fracture due to spinal tumor, myeloma or metastases; (6) previous history of spinal surgery; (7) incomplete radiological examinations; (8) loss to follow-up. The study was approved by the Ethics Committee of China-Japan Union Hospital of Jilin University and was in accordance with the Helsinki declaration. The author is not allowed to access information that can identify individual participants during or after data collection. Informed consent was obtained from all participants prior to enrollment.

### Surgical technique

**Figure 1:**
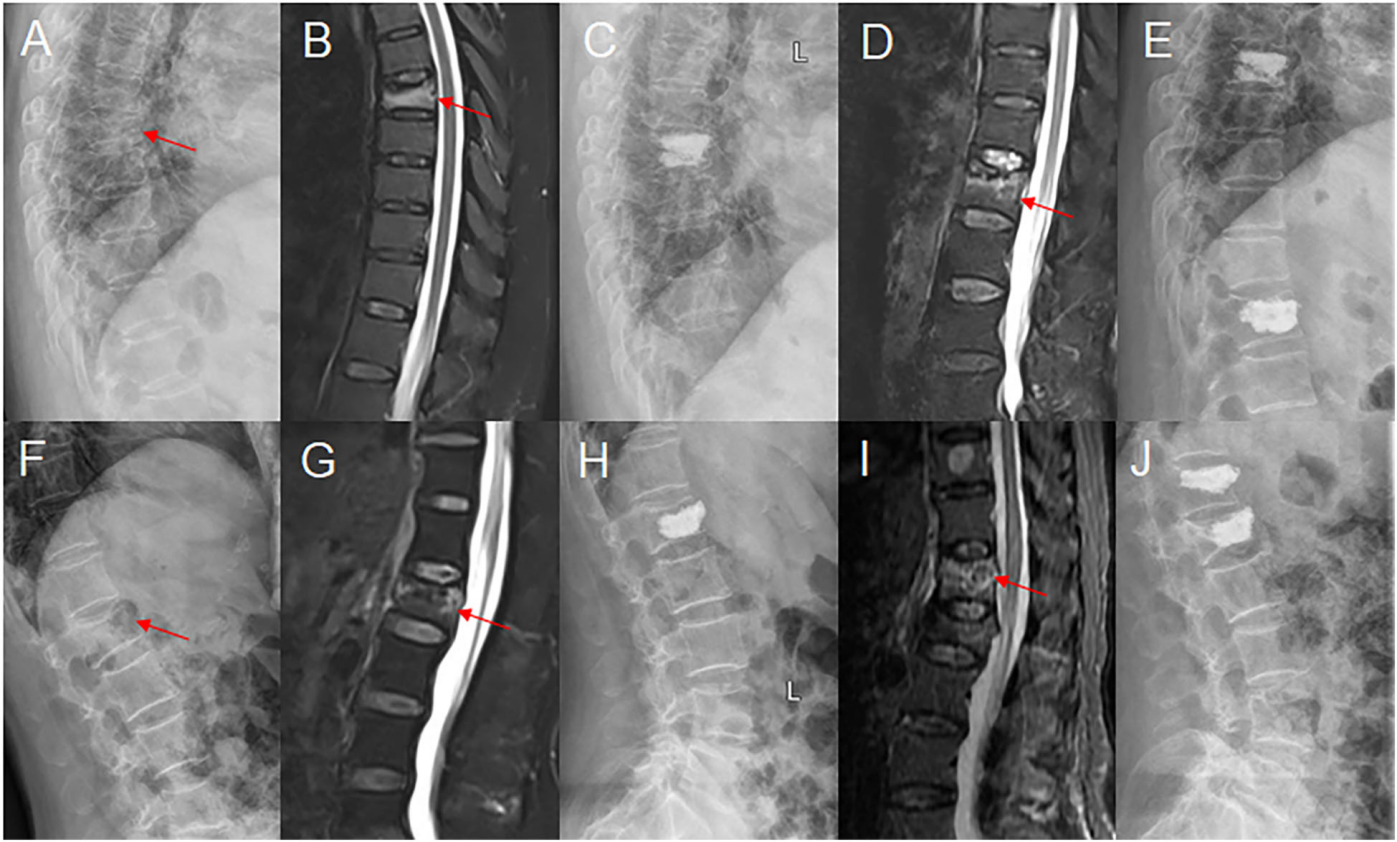
Two typical cases of SVF after PVP **(A-E)** and PKP **(F-J)**, respectively. **(A, B)** T7 fractured vertebrae was showed on X-ray and MRI. **(C)** T7 vertebrae treated with PVP was observed on X-ray. **(D)** MRI revealed T12 vertebrae fracture as SVF. **(E)** T12 vertebrae filled with bone cement was revealed on X-ray. **(F, G)** L1 fractured vertebrae was found on X-ray and MRI. **(H)** L1 vertebrae treated with PKP was observed on X-ray. **(I)** MRI revealed T12 vertebrae fracture as SVF. **(J)** T12 vertebrae filled with bone cement was showed on X-ray.

All the patients were placed on operative table in the prone position. And the PVP or PKP procedure was successfully performed under local anesthesia. The position of fractured vertebrae was determined with the guidance of fluoroscopic C-arm prior to a procedure. Subsequently, the insertion point of puncture needle was marked on the skin. Then, 1% lidocaine was slowly injected from the skin to the periosteum of the pedicle. Immediately, a puncture needle was inserted into the collapsed vertebrae through the pedicle of the vertebral arch, and the optical position of the puncture needle was confirmed under fluoroscopy. Subsequently, a 13-gauge bone biopsy needle cannula was advanced over the guide needle to establish a working channel, and positioned into the anterior third of the collapsed vertebral body near the midline. Similarly, its optimal position was determined with the help of fluoroscopic C-arm. In PKP procedure, there was an additional step where an inflatable tamp or balloon need to be inserted into the fractured vertebra so as to restore the high of collapsed vertebrae and correct of kyphosis deformity when it inflated. As a consequence, a working channel into the fractured vertebrae was established. Subsequently, the bone cement was loaded into a dedicated injection syringe and connected to the working channel. The cement, at a toothpaste-like consistency, was slowly and controllably injected into the collapsed vertebral body by rotating the plunger driver under fluoroscopic guidance. It was note that bone cement injection should be stopped immediately when bone cement leakage was noticed. The procedure ended when sufficient filling of the collapsed vertebrae was observed. Finally, the working channel was removed, and the incision was bandaged. After the procedure, all the patient needed to stay in bed for a few hours. All patients were performed with preoperative and postoperative radiological examinations (**Figures 1A-J**).

### Data collection

**Figure 2:**
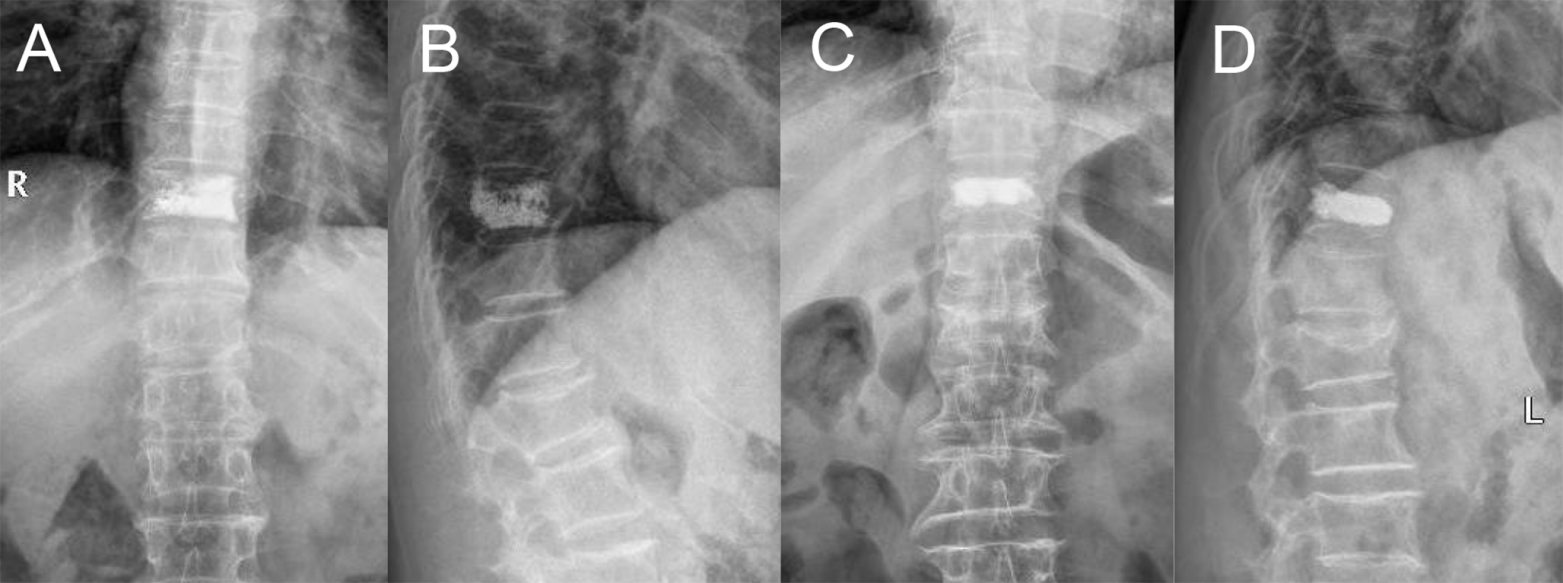
Bone cement distribution pattern on anterior and lateral radiography. **(A, B)** Trabecular type. **(C, D)** Compact type.

**Figure 3:**
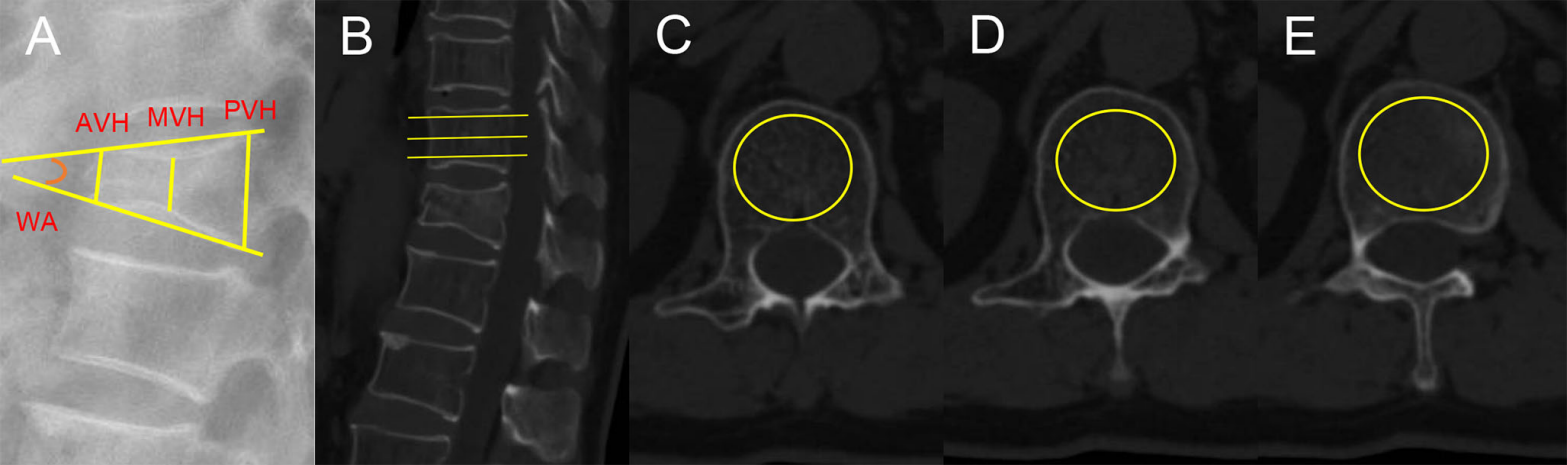
The measurement of radiographic data. **(A)** The AVH, MVH, PVH and wedge angle (WA) were measured on lateral X-ray. **(B-E)** HU value was measured on CT scanning.

Demographic variables, including age, BMI, hypertension, diabetes, history of smoking, history of drinking, bisphosphonate therapy, previous vertebral fracture history and duration of follow up, were documented from electronic medical records. Surgery-related parameters, such as surgical method, cement distribution pattern, bone cement volume, bone cement leakage and intradiscal cement leakage, were collected. Radiographic data, for example anterior vertebral height (AVH), middle vertebral height (MVH), preoperative degree of anterior vertebral compression (DAVC), preoperative degree of middle vertebral compression (DMVC), preoperative wedge angle, postoperative wedge angle, wedge angle correction, wedge angle correction rate and Hounsfield unit (HU) value, were measured on X-ray or computed tomography (CT). There were two surgical methods: PVP and PKP. Cement distribution pattern, including the trabecular type and the compact type, was determined on anterior and lateral radiography of spine (20). The trabecular type was defined when fractured vertebrae was filled with sponge-like cement, and the compact type was considered when collapsed vertebrae was filled with compact and solid cement (**Figures 2A-D**). Preoperative DAVC and DMVC were regarded as indicators of the degree of fractured vertebral compression. The measurement of preoperative DAVC and DMVC were based on a previous study (21). The AVH, MVH, and the posterior vertebral height (PVH) of the initial fractured vertebrae were measured on lateral radiography of spine (**Figure 3A**). The preoperative DAVC and DMVC were calculated using the formulas (AVH/PVH) × 100% and (MVH/PVH) × 100%, respectively. The wedge angle was defined as an angle that was formed by the superior endplate of the fractured vertebrae and the inferior endplate of the fractured vertebrae. Preoperative and postoperative wedge angle of the initial fractured vertebrae were also measured on lateral radiography of spine, with reference to Sadiqi’s method (**Figure 3A**) (21). The wedge angle correction was equal to the preoperative wedge angle minus the postoperative wedge angle. And the wedge angle correction rate was defined as a percentage that was derived by dividing the difference between the preoperative wedge angle and the postoperative wedge angle by the preoperative wedge angle. HU value was used to be an indicator of BMD, and it was measured by an elliptical region of interest on the axial CT images (22). It should be noted that CT scans were acquired using a General Electric 128-slice CT scanner (General Electric Company, USA) with the following parameters: tube voltage 120 kV, tube current 500 mA, and slice thickness 1 mm. The reconstruction kernel used was B35f. The HU value of the L1 vertebrae was derived from the average of measurements across three axial slices: inferior to the superior endplate, the mid-vertebral level, and superior to the inferior endplate (**Figures 3B-E**). The circular or elliptical ROI placement should exclusively include trabecular, strictly avoiding both cortex and any phantom calibration. It is note that HU value of T12 vertebrae or L2 vertebrae was used when L1 vertebrae fractured. All the radiological data were measured by two professional doctors.

### Statistical analysis

The SPSS Statistics Version 24 (IBM Corporation, Armonk, NY, USA) was used for statistical analysis of all data. Quantitative variables, including age, BMI, duration of follow up, bone cement volume, preoperative DAVC, preoperative DMVC, postoperative wedge angle, wedge angle correction, wedge angle correction rate and HU value, were expressed by means and standard deviations. And qualitative variables, such as hypertension, diabetes, history of smoking, history of drinking, bisphosphonate therapy, previous vertebral fracture history, surgical method, cement distribution pattern, bone cement leakage and intradiscal cement leakage, were described as frequencies with percentages. Univariate logistic regression analysis was performed to compare the differences between SVF group and control group in terms of demographic variables, surgery-related parameters and radiographic data. Among all variables, based on the results of univariate logistic regression analysis, those variables with the *P* value less than 0.05 analysis were further analyzed. Then, multivariate logistic regression analysis was used to identify the correlation between SVF and significant variables and further determine the independent risk factors of SVF following PVP or PKP for the treatment of single-level OVCF in postmenopausal women. *P*-value < 0.05 was considered statistically significant.

## Results

### Subjects’ characteristics of SVF group

A total of 528 postmenopausal women were enrolled in this present study, with the average age of 70.20 ± 8.32 years. And the average BMI of 528 patients was 23.10 ± 3.04 kg/m^2^. Of the 528 patients, 73 (13.83%) women who experienced SVF after PVP or PKP for initial single-level OVCF were assigned to SVF group. The average age and BMI of SVF group were 72.78 ± 8.09 years and 21.99 ± 2.76 kg/m^2^, respectively. In SVF group, 24 (32.9%) of 73 women had a history of previous vertebral fracture that was not treated with surgery (**Table 1**). Among 528 patients followed for a total of 10,082 person-months, the overall person-time incidence of SVF was 86.89 per 1000 person-years (**Table 2**). Further analysis revealed 40 cases of adjacent vertebral fracture (AVF), defined as re-fracture occurring in the segment adjacent to the initial fractured vertebrae, and 33 cases of non-AVF. Notably, the patients in the AVF group were significantly older than those in the non-AVF group (**Table 3**).

**Table 1:** Baseline characteristics and univariable regression analysis the variates between SVF group and control group.

Variable	All patients (n = 528)	Control group (n = 455)	SVF group (n = 73)	*P*
Age, years	70.20 ± 8.32	69.79 ± 8.29	72.78 ± 8.09	0.005
BMI, kg/m^2^	23.10 ± 3.04	23.28 ± 3.05	21.99 ± 2.76	0.001
Hypertension, n (%)	188 (35.6)	167 (36.7)	21 (28.8)	0.190
Diabetes, n (%)	108 (20.5)	95 (20.9)	13 (17.8)	0.546
Smoking, n (%)	40 (7.6)	32 (7.0)	8 (11.0)	0.243
Drinking, n (%)	6 (1.1)	4 (0.9)	2 (2.7)	0.187
Bisphosphonate therapy, n (%)	212 (40.2)	183 (40.2)	29 (39.7)	0.936
Previous fracture history, n (%)	86 (16.3)	62 (13.6)	24 (32.9)	< 0.001
Duration of follow up, months	19.09 ± 3.55	19.05 ± 3.58	19.34 ± 3.37	0.521
Surgical method, n (%)				0.307
PVP	367 (69.5)	320 (70.3)	47 (64.4)	
PKP	161 (30.5)	135 (29.7)	26 (35.6)	
Cement distribution, n (%)				0.572
Trabecular type	341 (64.6)	296 (65.1)	45 (61.6)	
Compact type	187 (35.4)	159 (34.9)	28 (38.4)	
Bone cement volume, ml	4.48 ± 1.04	4.52 ± 1.04	4.23 ± 0.99	0.029
Bone cement leakage, n (%)	115 (21.8)	100 (22.0)	15 (20.5)	0.784
Intradiscal cement leakage, n (%)	71 (13.4)	61 (13.4)	10 (13.7)	0.946
Preoperative DAVC, %	74.54 ± 13.78	74.79 ± 13.76	72.96 ± 13.93	0.293
Preoperative DMVC, %	64.90 ± 12.38	65.47 ± 12.33	61.35 ± 12.20	0.009
Postoperative wedge angle, °	7.44 ± 4.79	7.33 ± 4.70	8.15 ± 5.27	0.176
Wedge angle correction, °	3.07 ± 2.64	3.14 ± 2.71	2.63 ± 2.15	0.125
Wedge angle correction rate, %	31.21 ± 19.63	31.68 ± 19.61	28.27 ± 19.64	0.169
HU value	71.97 ± 27.05	74.75 ± 26.74	54.79 ± 22.37	< 0.001

SVF, subsequent vertebral fracture; BMI, body mass index; PVP, percutaneous vertebroplasty; PKP, percutaneous kyphoplasty; HU, Hounsfield unit.

**Table 2:** The overall person-time incidence of SVF.

SVF patients	All patients	Total person-months of follow-up for all patients	The overall person-time incidence of SVF
73	528	10082	86.89 per 1000 person-years

SVF, subsequent vertebral fracture.

**Table 3:** Baseline characteristics analysis of AVF group and non-AVF group.

Variable	SVF patients (n = 73)	Non-AVF group (n = 33)	AVF group (n = 40)	*P*
Age, years	72.78 ± 8.09	70.70 ± 8.05	74.50 ± 7.80	0.045
BMI, kg/m^2^	21.99 ± 2.76	22.20 ± 2.91	21.83 ± 2.66	0.572
Hypertension, n (%)	21	7 (21.2)	14 (35.0)	0.195
Diabetes, n (%)	13	6 (18.2)	7 (17.5)	0.940
Smoking, n (%)	8	2 (6.1)	6 (15.0)	0.224
Drinking, n (%)	2	1 (3.0)	1 (2.5)	1.000

SVF, subsequent vertebral fracture; BMI, body mass index; AVF, adjacent vertebral fracture.

### Univariate regression analysis of the variates between SVF group and control group

In the univariate logistic regression analysis, patients with SVF were older than those without SVF (*P* = 0.005), and BMI of SVF group was significantly lower than that of control group (*P* = 0.001). The variates, including previous fracture history (*P* < 0.001), bone cement volume (*P* = 0.029), preoperative DMVC (*P* = 0.009) and HU value (*P* < 0.001), significantly differed between the two groups. However, in the two groups, no significant differences were observed with respect to demographic variables, such as hypertension (*P* = 0.190), diabetes (*P* = 0.546), smoking (*P* = 0.243), drinking (*P* = 0.187), bisphosphonate therapy (*P* = 0.936) and duration of follow up (*P* = 0.521). In addition, there were no significant differences between the two groups in terms of surgery-related parameters, for example surgical method (*P* = 0.307), cement distribution (*P* = 0.572), bone cement leakage (*P* = 0.784) and intradiscal cement leakage (*P* = 0.946). Similarly, the two groups did not significantly differ with regard to radiographic data, for instance preoperative DAVC (*P* = 0.293), postoperative wedge angle (*P* = 0.176), wedge angle correction (*P* = 0.125) and wedge angle correction rate (*P* = 0.169) (**Table 1**).

### Multivariate regression analysis of associations between the variates and SVF

Only seven variables (age, BMI, previous fracture history, bone cement volume, preoperative DMVC and HU value) with a *P*-value < 0.05 were analyzed by the multivariate logistic regression analysis. Based on the results of the multivariate logistic regression analysis, age (OR = 1.009; 95% CI = 0.976-1.042, *P* = 0.610), bone cement volume (OR = 0.845; 95% CI = 0.654-1.091, *P* = 0.197), and preoperative DMVC (OR = 0.986; 95% CI = 0.966-1.006, *P* = 0.161) were not significantly associated with SVF after PVP or PKP. After adjusting for the confounding factors, BMI (OR = 0.905; 95% CI = 0.829-0.987, *P* = 0.025) and HU value (OR = 0.977; 95% CI = 0.966-0.988, *P* < 0.001) was inversely associated with risk of SVF, and previous fracture history (OR = 1.899; 95% CI = 1.046-3.449, *P* = 0.035) significantly increased the risk of SVF in postmenopausal women who treated with PVP or PKP for single-level OVCF (**Table 4**).

**Table 4:** Multivariate regression analysis of associations between the variates and SVF.

Variable	B	SE	Wald	OR (95% CI)	*P*
Age, years	0.009	0.017	0.260	1.009 (0.976-1.042)	0.610
BMI, kg/m^2^	-0.100	0.045	5.043	0.905 (0.829-0.987)	0.025
Previous fracture history, n (%)	0.642	0.304	4.444	1.899 (1.046-3.449)	0.035
Bone cement volume, ml	-0.169	0.131	1.667	0.845 (0.654-1.091)	0.197
Preoperative DMVC, %	-0.014	0.010	1.965	0.986 (0.966-1.006)	0.161
HU value	-0.023	0.006	15.693	0.977 (0.966-0.988)	< 0.001

SVF, subsequent vertebral fracture; BMI, body mass index; HU, Hounsfield unit; B, unstandardized B coefficient; S.E., standard error; OR, odds ratio; CI, confidence interval.

## Discussion

In this retrospective cohort study, the results showed that the cumulative incidence of SVF after PVP or PKP for the treatment of single-level OVCF in postmenopausal women was 13.83%. In a network meta-analysis of 23 randomized controlled trials with 2,838 patients, Essibayi et al. (23) compared the risk of adjacent-level fractures after vertebroplasty and kyphoplasty after OVCF and found that the incidence of SVF for both PVP and PKP was 8%, with no significant difference between the two treatments. The higher incidence of SVF observed in this study may be attributed to differences in the study population and methodology. This retrospective study exclusively included postmenopausal women with severe osteoporosis, who appear to have a higher propensity for SVF, whereas the referenced RCT enrolled a broader patient population. Furthermore, the follow-up period of at least 12 months in this study likely captured more SVF events compared to the RCT. Additionally, the finding of no significant difference in the SVF incidence between PVP and PKP procedures was consistent with the study by Essibayi et al. (23), which helped provide evidence-based guidance for the selection of these two surgical techniques in clinical practice.

Based on the results of the univariate and multivariate logistic regression analysis, the findings of this present study concluded that low BMI was associated with an increased risk of SVF, previous fracture history was an independent risk factor of recurrent fracture, and low BMD significantly increased the recurrence of fracture in postmenopausal women who were treated with PVP or PKP for single-level OVCF. To the best of our knowledge, this is the first systematic and comprehensive study to investigate the risk factors of SVF following initial PVP or PKP for the treatment of single-level OVCF in postmenopausal women.

The result of our study demonstrated that low BMI increased risk of SVF following PVP or PKP in postmenopausal women. It is well known that postmenopausal women with low BMI are at increased risk for fractures. A prospective cohort study that included 52,629 postmenopausal women from ten countries determined the relationship between BMI and osteoporotic fracture in postmenopausal women. The result of study indicated that BMI was inversely associated with the incidence of clinical vertebral fracture in postmenopausal women (24). An observational study of 925,345 individuals revealed that high BMI was associated with a decreased incidence of fracture in postmenopausal women (25). High BMI seems to be a protective factor of fracture. There are some possible mechanisms regarding high BMI and low risk of fracture. Firstly, higher body mass increases strain on the bone, which could improve BMD to some extent (26). Another mechanism is that more adipose tissue promotes an increase of estrogens, helping in the preservation of BMD (27). Thirdly, more fat mass decreases the impact force of a fall from height, which reduces the incidence of fracture (28). Therefore, interventions regarding low BMI should be recommended to reduce the risk of SVF following PVP or PKP in postmenopausal women.

According to the result of the multivariate regression analysis, previous vertebral fracture significantly increased the rate of SVF in postmenopausal women after PVP or PKP for single-level OVCF. It is well established that prevalent vertebral fracture is a risk factor of new vertebral fracture. One study with a 25-year follow-up period investigated the incidence and risk factors for vertebral fracture in women and men. The result of the study demonstrated that prevalent vertebral fracture was related to the incidence of new vertebral fractures, only limiting to individuals with moderate to severe prevalent vertebral fracture (29). In a longitudinal cohort study involving 9,704 white women over 15 years of follow-up, Cauley et al. (29) concluded that prevalent vertebral fracture significantly enhanced the risk of new incident vertebral fracture. However, when it comes to postmenopausal female patients, there are few related studies. An observational study that included 2,725 postmenopausal women explored the risk of new vertebral fracture after a vertebral fracture. The conclusion of this study revealed that the presence of prevalent vertebral fracture increased the risk of new vertebral fracture after a vertebral fracture with the next year (30), consisting with our result of the present study. It should be noted that primary vertebral fracture need be identified as early as possible. More importantly, postmenopausal women with primary vertebral fracture should receive more attention and effective interventions to reduce the occurrence of secondary vertebral fractures.

The finding of our study showed that low BMD was associated with the incidence of SVF in postmenopausal women after PVP or PKP. It is well accepted that low BMD is correlated with an increased risk of vertebral fracture. BMD is considered a quantitative method for the severity of osteoporosis. The risk of new vertebral fracture increased as osteoporosis worsened. Previous study suggested that low bone mass could predict the occurrence of new fracture (31). In a retrospective cohort study, Lu et al. (19) investigated the potential risk factors of SVF after vertebroplasty. The result demonstrated that low BMD was an independent risk factor of SVF following PVP. A literature review that included 24 observational studies determined the risk factors of new vertebral compression fracture following PVP. The authors concluded that lower BMD was regarded as a strong-evidence risk factor of new vertebral fracture in patients following PVP (32). A retrospective study, only including postmenopausal women, revealed the strong association between low BMD and the risk of new vertebral fracture in who were treated with PVP for OVCF (33), which was in line with our findings of the present study. In our study, the HU value that was measured on CT scanning rather than the T-score obtained from the dual energy X-ray absorptiometry is used as an indicator of BMD to evaluate osteoporosis. It is important to note that a number of anti-osteoporosis measures that are effective in increasing BMD should be applied to postmenopausal women with osteoporosis, especially those treated with PVP or PKP, so as to reduce or even prevent the occurrence of SVF following percutaneous vertebral augmentation.

This study conducted a subgroup analysis of patients with SVF. The age of patients with AVF was significantly higher than that of patients with non−AVF, whereas no significant differences were observed between the two groups in other aspects. This important finding can provide evidence−based guidance for the preliminary diagnosis of SVF. Additionally, after percutaneous augmentation for non−AVF, the treated vertebra together with the primarily augmented vertebra may form a sandwich vertebra. According to the literature (13), the fracture rate of sandwich vertebrae was 21.3%, of which 55.5% occurred within the first year, whereas the incidence of sandwich vertebrae fracture was not higher than that at other adjacent segments. Therefore, clinicians should focus not only on SVF in general but also pay further attention to AVF, non−AVF, and sandwich vertebral fractures.

There exist some limitations that should be noted in this present study. Firstly, this study only includes postmenopausal women patients who underwent PVP or PKP for initial single-level OVCF, which cannot be homogenized to all postmenopausal women, even men. Of course, the incidence rate of SVF is underestimated. Secondly, some variables are not clearly described in this present study. For instance, the number of previous vertebral fracture is not present in detail. Finally, our study is a retrospective study in a single-center, which may lead to the bias of selection. Moreover, we cannot assure the causal relationship between risk factors and SVF based on this retrospective design. It is necessary that prospective randomized controlled, large sample size, and multicenter trials are conducted to better investigate the risk factors of SVF following PVP or PKP in the future.

## Conclusions

In our present study, BMI, previous vertebral fracture history as well as BMD were independent risk factors of new vertebral fracture and significantly increased the incidence of SVF in postmenopausal women who were treated with PVP or PKP for single-level OVCF. Our findings provide guidance to orthopedic surgeons in making decisions. We suggested that those patients with risk factors should be identified as early as possible, and some effective intervention measures should be timely recommended and implemented to reduce or even avoid the occurrence of SVF.

## Data Availability

The original contributions presented in the study are included in the article/supplementary material. Further inquiries can be directed to the corresponding author.
